# Exploration of Lung Cancer-Related Genetic Factors *via* Mendelian Randomization Method Based on Genomic and Transcriptomic Summarized Data

**DOI:** 10.3389/fcell.2021.800756

**Published:** 2021-12-06

**Authors:** Nitao Cheng, Xinran Cui, Chen Chen, Changsheng Li, Jingyu Huang

**Affiliations:** ^1^ Department of Thoracic Surgery, Zhongnan Hospital, Wuhan University, Wuhan, China; ^2^ School of Computer Science and Technology, Harbin Institute of Technology, Harbin, China; ^3^ Department of Biological Repositories, Zhongnan Hospital, Wuhan University, Wuhan, China

**Keywords:** lung cancer, susceptibility genes, GWAS, eQTL, SMR

## Abstract

Lung carcinoma is one of the most deadly malignant tumors in mankind. With the rising incidence of lung cancer, searching for the high effective cures become more and more imperative. There has been sufficient research evidence that living habits and situations such as smoking and air pollution are associated with an increased risk of lung cancer. Simultaneously, the influence of individual genetic susceptibility on lung carcinoma morbidity has been confirmed, and a growing body of evidence has been accumulated on the relationship between various risk factors and the risk of different pathological types of lung cancer. Additionally, the analyses from many large-scale cancer registries have shown a degree of familial aggregation of lung cancer. To explore lung cancer-related genetic factors, Genome-Wide Association Studies (GWAS) have been used to identify several lung cancer susceptibility sites and have been widely validated. However, the biological mechanism behind the impact of these site mutations on lung cancer remains unclear. Therefore, this study applied the Summary data-based Mendelian Randomization (SMR) model through the integration of two GWAS datasets and four expression Quantitative Trait Loci (eQTL) datasets to identify susceptibility genes. Using this strategy, we found ten of Single Nucleotide Polymorphisms (SNPs) sites that affect the occurrence and development of lung tumors by regulating the expression of seven genes. Further analysis of the signaling pathway about these genes not only provides important clues to explain the pathogenesis of lung cancer but also has critical significance for the diagnosis and treatment of lung cancer.

## Introduction

Pulmonary carcinoma is the leading cause of cancer deaths in the world. According to the Global Cancer Statistics report, there were about 2 million newly detected lung cancer cases worldwide in 2018, accounting for 11.6% of all tumors (Bray et al., 2018). Deaths from lung cancer worldwide were roughly 1.7 million, accounting for 18.4% of tumor-related deaths. Lung cancer is a malignant disease resulting from malignant changes of bronchial and alveolar epithelial cells. Lung cancer is divided into two categories: non-small cell lung cancer (NSCLC) and small cell lung cancer (SCLC). Of all diagnosed cases of lung cancer, NSCLC accounts for about 85% (Oser et al., 2015). Based on the characteristics of lung carcinoma, non-small cell lung cancer is also split into three: pulmonary adenocarcinoma, Lung squamous cell carcinoma, and large cell lung cancer. Although SCLC is a small proportion, it grows and spreads faster than NSCLC and tends to metastasize early in the disease (Zakaria et al., 2015). Bearing these facts in mind, the therapy of lung cancer faces a serious challenge.

It is immediately unclear how pulmonary carcinoma develops. As shown in many kinds of research, the occurrence of lung cancer can be driven by multiple factors including genetic and environmental factors. In particular, SCLC is generally related to lifestyle and environment (Rudina et al., 2021). Besides external environmental factors, genetic factors also play an important role in the occurrence of lung cancer. For example, the incidence of lung adenocarcinoma represents more than 55% of NSCLC, and it is more appropriate to many women and non-smokers (Barr Kumarakulasinghe et al., 2015). These people tend to carry gene mutations. The common mutated genes contain EGFR, ALK, ROS1 gene, and so on (Lamberti et al., 2020). In a word, exploring genetic factors has critical implications for understanding the pathogenic mechanism of pulmonary cancer.

In recent decades, many genetic studies have been utilized to determine the genetic factors of lung cancer. Genome-wide association studies (GWAS) as a hot research tool at present have been applied in lung cancer, and have successfully identified many genetic susceptibility sites (Landi et al., 2009). GWAS perform genotype on several hundreds of thousands of Single Nucleotide Polymorphisms (SNPs) according to the comparison of a large number of lung cancer cases and healthy controls, and then bioinformatics techniques and statistical methods were employed to compare the frequency diversities of each SNP between the two groups (Ko and Urban, 2013). In the results of GWAS analysis, most important SNPs are located in non-coding regions, thus it is difficult to directly explore the regulatory mechanism of these loci (Rojano et al., 2016). Therefore, although we have identified many susceptibility sites associated with lung tumors, it remains unknown about the role of lung cancer pathogenesis-related genes or DNA elements.

Accordingly, this research adopted the method of multi-omics analysis to consolidate various sources of data in an attempt to identify genes related to lung cancer incidence. With the development of high-throughput omics platforms, multi-omics analysis is one of the most important and widely used in biomedical research (Tanaka et al., 2020; Tarwadi et al., 2020; Zhao et al., 2021; Zhao, 2021). As these are kind of hard to describe diseases with complex etiologies by a single omics analysis, the multi-omics research method is developed to provide more evidence for the disease pathogenesis and dig out candidate key factors in depth. Data from different omics sources such as genomics, transcriptomics, and proteomics can be integrated through machine learning or statistical methods for purpose of underlying biological mechanisms (Zhao et al., 2020; Reel, 2021). In this study, Summary data-based Mendelian Randomization (SMR) was used as a statistical method for multi-omics analysis.

SMR was conducted to investigate the pathogenic genes of lung cancer. SMR analysis is an improved model based on Mendelian randomization study that uses genetic variation as a tool to predict the effects of simulated exposure factor on disease using gene-disease causal inference (Zhu et al., 2016). Compared with the general instrumental variable models, the MR model can significantly reduce the estimation bias caused by measuring error. In the MR model, genetic variants as an instrumental variable have to satisfy three core assumptions. The three core assumptions consist of the relevance assumption, the independence assumption, and the exclusion restriction assumption (Hartwig and Davies, 2016). The relevance refers to a robust correlation between genetic variants and exposure factors. The independence assumption represents the independence of genetic variation and confounding factors affecting the expose-outcome relationship (Zhao et al., 2019). The exclusion restriction states that genetic variation can affect outcomes only through exposure factors and not through other pathways. The violation of any of the core assumptions may distort the statistical inference results of MR analysis, leading to incorrect inferences of causality.

However, the existence of linkage disequilibrium (LD) or pleiotropy of instrumental variables can be contrary to the exclusion restriction. Once two genes are not completely independent, they will show some degree of linkage, which is called linkage imbalance. Pleiotropy is the phenomenon that one gene can affect more than one trait. Due to the coordinate positions in the genome between SNPs and the complicated biological effects between genetic variants and traits, LD and pleiotropy are often unavoidable (Wang, 2021). Therefore, to soften the influence of the exclusivity hypothesis, a variety of modified MR methods, such as Mendelian Randomization-Egger regression (MR-Egger), Inverse Variance Weighted (IVW), Mode-Based Estimate (MBE) method, have been proposed in recent years. These methods only consider the analytical error because of pleiotropy. SMR method also takes into account the LD between instrumental variables to decrease the false positive rate. In addition, there are fewer requirements for the summary data of GWAS and eQTL, and the sample size in SMR analysis. As a result, we adopted this approach because of its wide applicability and high accuracy.

In this research, we integrated lung cancer GWAS data and eQTL data to recognize functional genes and regulatory elements in pulmonary carcinoma. Statistical analysis of the relationship between a single SNP and gene expression is referred to as eQTL analysis (Shabalin, 2012). Namely, the expression of a gene is affected by a single SNP, this variable site is considered to be an eQTL locus. Since the subjects of both GWAS and eQTL were SNPs, SMR analysis of overlapping mutants presented in GWAS and eQTL data could reveal lung cancer related-genes. The discovery of these genes could effectively support the design of targeted drugs and precise treatment of lung cancer.

## Materials and Methods

### Acquisition of GWAS Summary Data

We downloaded two available GWAS summary data of lung cancer from the GWAS Catalog website. One of the GWAS datasets was obtained from the meta-analysis of the United Kingdom Biobank (UKB) and the Kaiser Permanente Genetic Epidemiology Research on Adult Health and Aging (GERA) (Rashkin et al., 2020). The UKB study was composed of 502,611 British people aged 40 to 69 at the time of recruitment. Study participants provided biological samples as well as detailed information on lifestyle and health-related factors. GERA participants were members of the Adult Kaiser Permanente Northern California (KPNC) Health Plan. The subjects of this study included 102,979 participants selected for their genotyping. Cancer cases in the UKB were identified on the basis of information provided by various national cancer registries that gathered data from hospitals, nursing homes, and so on. GERA cancer cases group were classified by using diagnoses from the KPNC cancer registry. The control group for the UKB and GERA studies was limited to individuals in the study who did not have any history of lung cancer in the relevant registries.

The other GWAS summary data was collected from the analysis of the BioBank Japan Project (BBJ) data (Sakaue, 2021). BBJ is a prospective biobank that collaboratively collects the DNA and serum samples of 200,000 Japanese participants from 12 medical institutions in Japan. All study participants were diagnosed with one or more of 47 target diseases by doctors. In this study, 178,726 participants of Japanese ancestry were selected from all participants. The researchers used a sample of the cohort without a given lung cancer diagnosis as a control group.

### Collection of eQTL Summary Data

Four eQTL data containing three blood eQTL data and one lung eQTL data were applied in our research. Two of eQTL data were obtained by sequencing whole peripheral blood mRNA of 2,116 healthy adults from four Dutch cohorts (Zhernakova et al., 2017). The difference between the two datasets was that one described 23,060 eQTL effects of gene level, the other included 21,888 eQTL effects of exon level. The remaining two of eQTL data were downloaded from the GTEx website. Since eQTL varies in different tissues or cells, we selected one lung eQTL dataset and one blood eQTL dataset to perform the analysis. Lung and blood eQTL datasets stored eQTL information about 25,245 genes and 20,049 genes, respectively.

### SMR Analysis Through Lung Cancer GWAS and eQTL Summary Data

**FIGURE 1:**
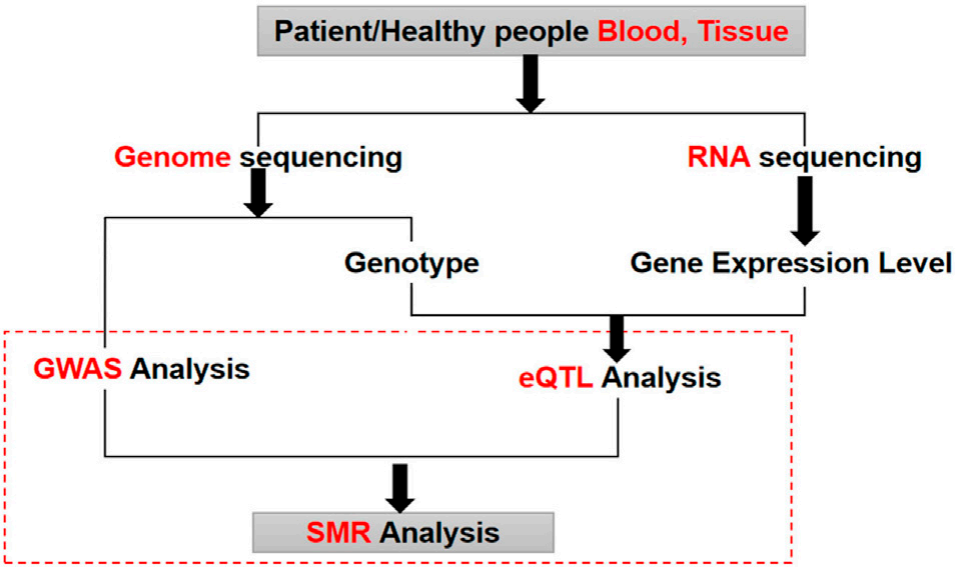
The process of GWAS and eQTL conjoint analysis. GWAS analysis and eQTL analysis are two different types of omics analysis. Using SMR method can discover the biological mechanism behind lung cancer by combining these two different omics data.

The research adopted the SMR analysis method to perform the conjoint analysis of eQTL and GWAS (Figure 1). Prior to compute SMR, we first need to find overlapped SNPs of GWAS and eQTL and generate a new dataset containing all overlapping SNPs information. Then the GWAS and eQTL data of new datasets were standardized by Z Score. We can calculate the Z value based on the Beta (OR) value and one of the *p* values or the SE value. The core algorithms of eQTL and GWAS are based on linear regression. Therefore, the Beta/OR value of GWAS and eQTL represents the value of the regression coefficient. SE value refers to the standard error of regression coefficient. The *p* value means the value of whether the association is significant. We listed two calculation formulas of the Z value below (Eq. 1; Eq. 2).

**FIGURE 2:**
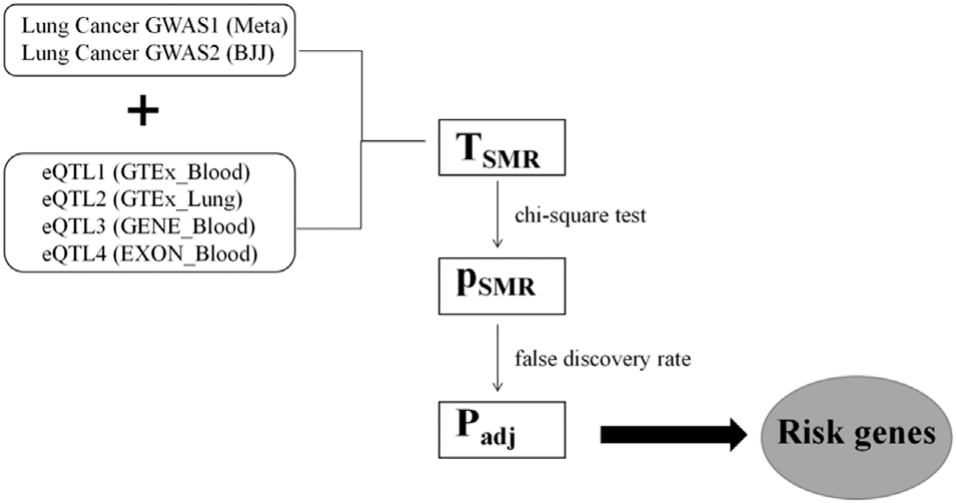
The process of GWAS and eQTL conjoint analysis. The two GWAS datasets of lung cancer were cross-analyzed with four groups of eQTL datasets. Significance of gene expression was found after T_SMR_ calculation, chi-square test, and FDR correction.

The Z values of GWAS and eQTL were used to calculate the T_SMR_ values according to Eq. 3 based on the Delta algorithm (Figure 2). The T_SMR_ values were output as P_SMR_ values by chi-square test. As False Discovery Rate (FDR) method can control false positive events by correcting the *p* values, we used FDR to adjust the P_SMR_ values, resulting in obtaining the P_adj_ values. The P_adj_ value of 0.05 was taken as the threshold of statistical significance, which means that the genes corresponding to P_adj_ less than 0.05 could be regarded as lung cancer-related pathogenic genes found in this study.

c=qnorm(1−p÷2); Beta>0, c=Z Score, Beta<0; c=−Z Score (1)

Z Score=Beta÷SE (2)

$$T_{SMR\approx \frac{{{Z_{GWAS}}^{2}\times Z_{eQTL}}^{2}}{Z_{GWAS}^{2}+Z_{eQTL}^{2}}}$$ (3)

## Results

### Discovery of the Overlapping SNPs Between GWAS and eQTL

**FIGURE 3:**
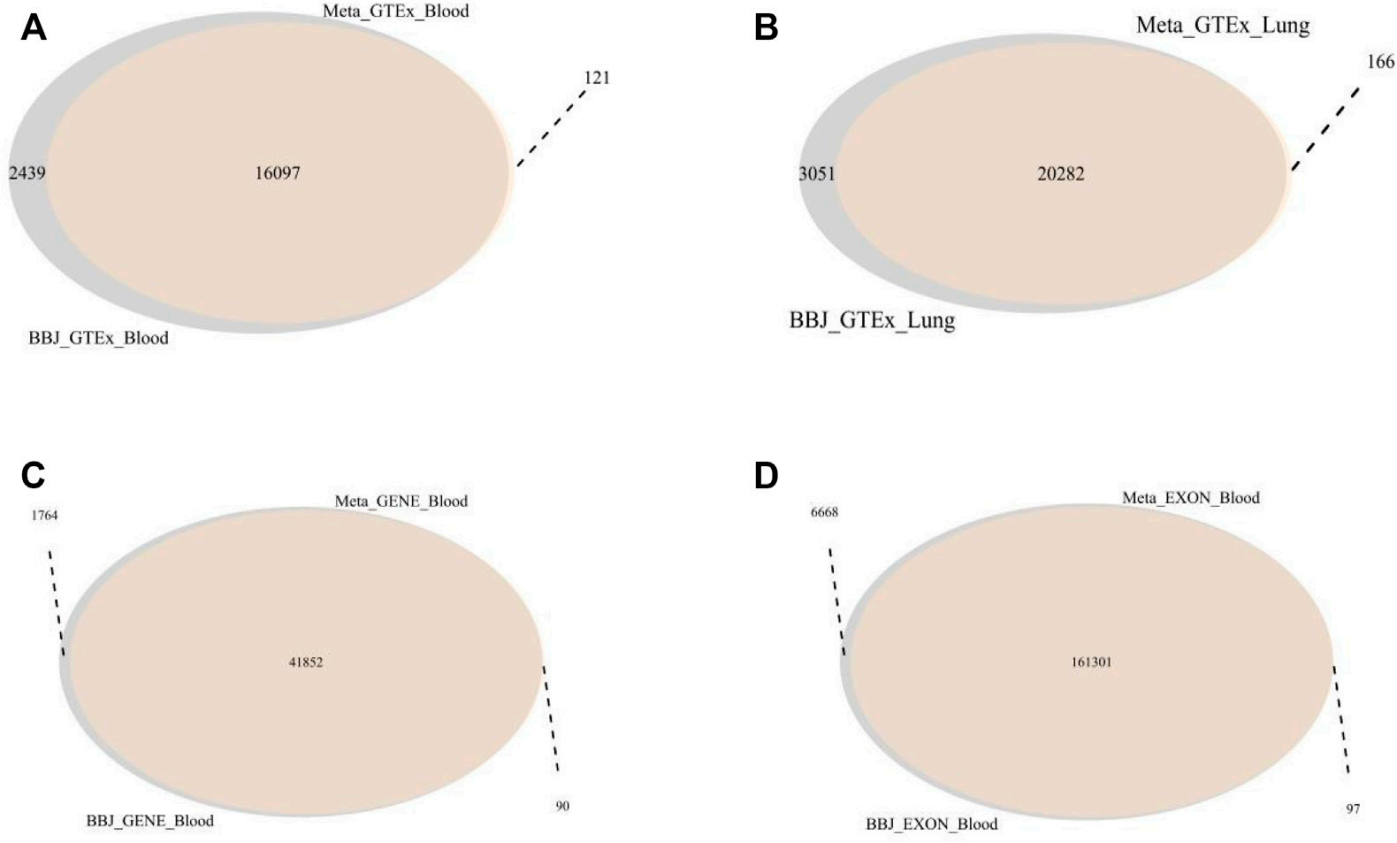
The overlapping genes between two different SMR datasets. **(A)** The overlap genes of Meta_GTEx_Blood and BBJ_GTEx_Blood. 16,097 genes were overlapped. **(B)** The overlap genes of the Meta_GTEx_Lung and BBJ_GTEx_Lung. There were 20,282 overlapped genes. **(C)** The overlap genes of Meta_GENE_Blood and BBJ_GENE_Blood. These two datasets have 41,852 identical genes. **(D)** the overlap genes of Meta_EXON_Blood and BBJ_EXON_Blood. These two datasets have 161,301 identical genes.

In this SMR analysis, the first step was to find the same SNPs between one of the GWAS datasets and one of the eQTL datasets and generate a new SMR dataset containing the details of all the overlapping SNPs (Table 1). We performed four SMR analyses by using GWAS summary data from a large meta-analysis to examine the association between lung cancer and gene expression probes from four eQTL data. The number of SNPs found in Meta_Blood_Exon data was higher than the other three data, with nearly a hundred thousand SNPs and more than twenty thousand genes found (Figure 3). The remaining three SMR datasets contained 10,000–20,000 SNPs and gene overlaps. In addition, GWAS data collected from the Japanese populations was also conducted four SMR analyses with different eQTL data. The overlap analysis results show that both BBJ_GTEx_Blood and BBJ_GTEx_Lung contained about 20,000 SNPs and genes. BBJ_GENE_Blood and BBJ_EXON_Blood included over 20,000 genes and thirty thousand SNPs and a hundred thousand SNPs, respectively.

**TABLE 1:** The new dataset list for SMR analysis. Eight SMR datasets were created for this analysis.

GWAS	eQTL	SMR	GWAS	eQTL	SMR
Meta	GTEx_Blood	Meta_GTEx_Blood	BBJ	GTEx_Blood	BBJ_GTEx_Blood
	GTEx_Lung	Meta_GTEx_Lung		GTEx_Lung	BBJ_GTEx_Lung
	GENE_Blood	Meta_GENE_Blood		GENE_Blood	BBJ_GENE_Blood
	EXON_Blood	Meta_EXON_Blood		EXON_Blood	BBJ_EXON_Blood

### Identification of Genes Associated With Lung Carcinoma

**FIGURE 4:**
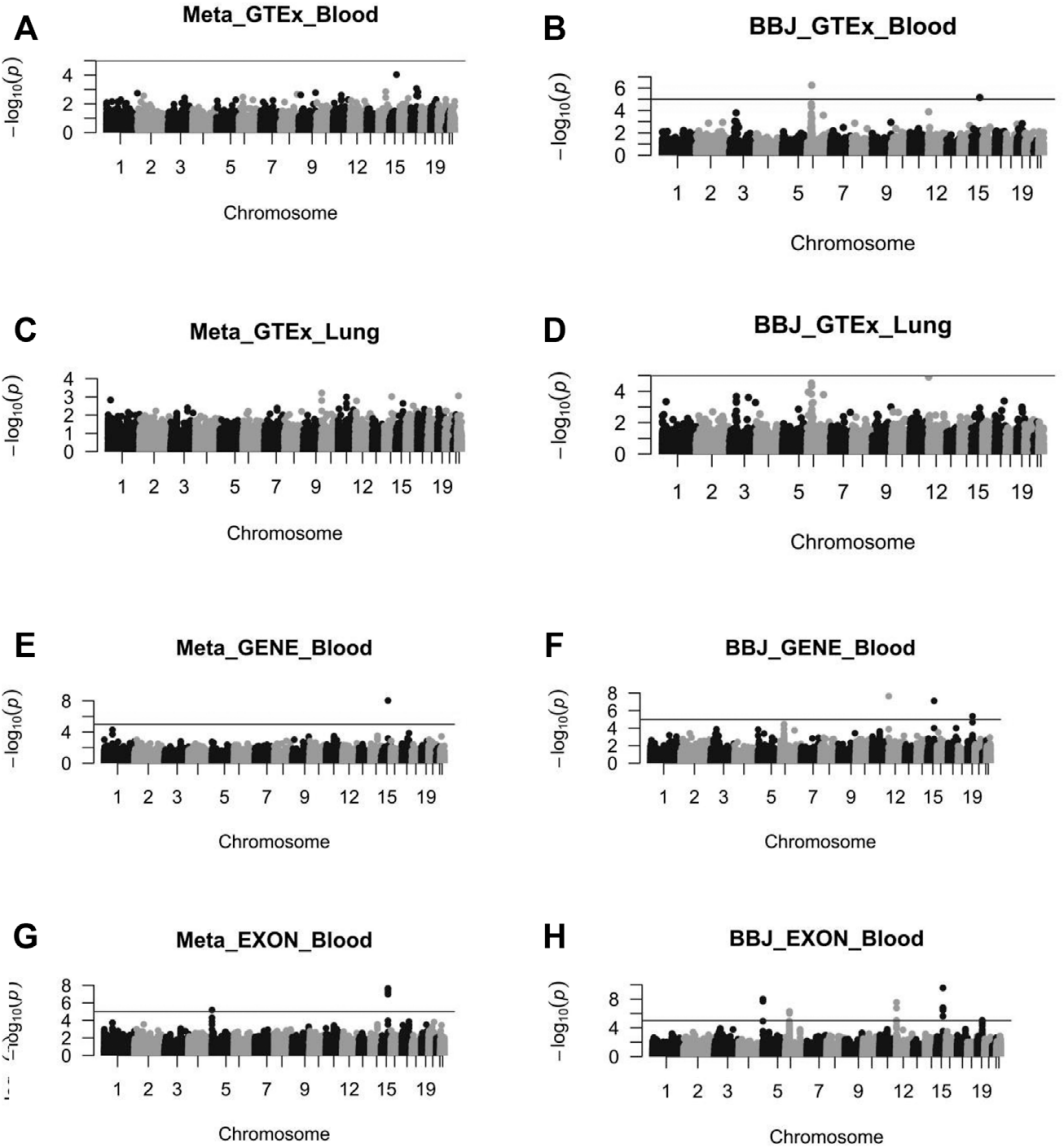
The Manhattan diagram of these eight SMR analysis results. **(A)** the SMR analysis results of Meta_GTEx_Blood. **(B)** the SMR analysis results of BBJ_GTEx_Blood. **(C)** the SMR analysis results of Meta_GTEx_Lung. **(D)** the SMR analysis results of BBJ_GTEx_Lung. **(E)** the SMR analysis results of Meta_GENE_Blood. **(F)** the SMR analysis results of BBJ_GENE_Blood. **(G)** the SMR analysis results of Meta_EXON_Blood. **(H)** the SMR analysis results of BBJ_EXON_Blood.

The Z values of GWAS and eQTL were calculated by the T_SMR_ formula. After the Chi-squared test, T_SMR_ values were transferred to P_SMR_ (Figure 4). Then we performed FDR on P_SMR_ to obtain P_adj_ values. Finally, we filtered P_adj_ values not less than 5%. As a result, two Padj values corresponding to different SNP susceptibility loci (rs8042849 and rs931794) were screened out (Table 2). These two SNP loci might regulate gene expression and hence cause the occurrence and development of cancer. Both loci were relevant to the expression of PSMA4 gene.

**TABLE 2:** The list of discovered lung cancer causative genes by the SMR analysis. This list showed the significant SNPs and genes found in different datasets, and the corresponding value of P_SMR_ and P_adj_ calculated.

SMR datasets	Significant SNPs	P_SMR_ value	P_adj_ value	Gene name
Meta_GENE_Blood	rs8042849	$9.17\times 10^{-9}$	0.0004	PSMA4
Meta_EXON_Blood	rs931794	$1.06\times 10^{-7}$	0.0029	PSMA4
	rs8042849	$2.44\times 10^{-8}$	0.0013	PSMA4
BBJ_GTEx_Blood	rs9274510	$5.70\times 10^{-7}$	0.0106	HLA-DQB1
BBJ_GENE_Blood	rs147560086	$2.26\times 10^{-8}$	0.0010	RAD52
	rs8042849	$7.81\times 10^{-8}$	0.0017	PSMA4
BBJ_EXON_Blood	rs31487	$1.96\times 10^{-8}$	0.0011	CLPTM1L
	rs147560086	$2.77\times 10^{-8}$	0.0012	RAD52
	rs12592111	$2.40\times 10^{-6}$	0.0288	IREB2
	rs8042849	$1.66\times 10^{-7}$	0.0040	PSMA4
	rs12822733	$1.74\times 10^{-7}$	0.0037	RAD52
	rs931794	$2.70\times 10^{-10}$	$4.53\times 10^{-5}$	PSMA4
	rs402710	$9.79\times 10^{-9}$	0.0008	CLPTM1L
	rs9274564	$5.15\times 10^{-7}$	0.0072	HLA-DQB1
	rs9274564	$5.14\times 10^{-7}$	0.0079	HLA-DRB9
	rs28755305	$9.50\times 10^{-7}$	0.0123	HLA-DQB2

The analysis of four SMR datasets from Asian populations identified a total of 10 SNP significant sites, most of which were repeatedly identified in these SMR analyses. The expression of seven genes was influenced by these 10 SNPs. The seven genes were comprised of HLA-DQB1, CLPTM1L, RAD52, IREB2, PSMA4, HLA-DRB9, and HLA-DQB2. According to the results, PSMA4 was recognized again. Furthermore, no significant susceptibility sites were found in the association analysis of GWAS data and lung eQTL data.

### Gene Function Analysis

To further explain the potential regulatory mechanism of gene expression on lung cancer susceptibility, we conducted the analysis of gene function and involved biological pathways on these seven genes on the Kyoto Encyclopedia of Genes and Genomes (KEGG) database. According to the analysis results, PSMA4 is an essential subunit of the proteasome protein. The proteasome is a large protein complex and is the key to regulating cell function cellular functions, such as cell differentiation, cell cycle (Voutsadakis, 2017). Many studies have shown that the proteasome plays an important role in the development and progression of various tumors, including lung cancer. In addition, three of the seven genes were the members of the Major Histocompatibility Complex (MHC) gene family. Human Leukocyte Antigen (HLA) stands for human MHC, The HLA gene family is divided into three subgroups, which encode three types of molecules: MHC class I molecules, MHC class II molecules, and MHC class III molecules. These three genes were of the MHC class II. When the external invaders are engulfed and processed by lysosome to be fragmented, the MHC class II molecules bind to these fragments and present them on the cell surface for T cells to recognize. The study indicates that HLA class II genes on tumor cells impact tumor immunogenicity (Chen et al., 2019).

Moreover, the CLPTM1L gene can encode cleft lip and palate transmembrane protein 1-Like protein. Recent research shows that the SNPs of CLPTM1L were associated with many human malignancies, especially in some highly aggressive and metastatic cancers (Shete et al., 2020). RAD52 function on the recombination repair of double strand breaks (DSB) is important for maintaining chromosome integrity. Based on the cause of cell death by DSB unrepair, cancer cells have a higher DSB burden, and hence the relationship between DSB and RAD52 can be exploited for cancer treatment (Trenner and Sartori, 2019). IREB2 gene can encode an iron-responsive element-binding protein that is an RNA-binding protein and can bind to iron response elements (IRES) for the regulation of the translation and stability of mRNAs in cells. Variants in the gene have been linked to lung cancer and chronic obstructive pulmonary disease (COPD). These results further proved this inference of our analysis. Accordingly, the analysis of the relationship between these genes and lung cancer can provide an important theoretical basis for revealing the genetic mechanism behind lung cancer.

## Discussion

We applied multi-omics analysis to integrate two of the GWAS datasets and four of eQTL datasets based on the SMR method, resulting in the functionally important genes related-lung cancer being found. SMR research can facilitate more accurate and effective identification of the potential causal relationship between exposure and outcome, for disease risk factors had a further understanding. Once risk factors have been successfully discovered, they can be prevented or targeted for cancer treatment. By analyzing the causal genes associated with lung cancer, we recognized seven genes associated with lung cancer, suggesting a possible mechanism for the development of lung cancer by altering the expression of risk genes.

With the analysis of these SMR datasets, the PSMA4 gene was repeatedly identified. It has been reported that down-regulation of proteasome subunit encoding gene PSMA4 reduces proteasome activity (Wang et al., 2007). The proteasome is a large protein complex that can degrade various cellular proteins rapidly and timely. The proteasome is responsible for regulating many cellular processes, including transcription, cell cycle progression, and apoptosis (Cardoso et al., 2004). Proteasome dysfunction leads to many diseases including cancer. Drugs that inhibit proteasome activity directly affect the susceptibility of lung cancer by regulating cell proliferation and apoptosis. Additionally, PSMA4 is involved in cancer cell proliferation, also its polymorphisms have been shown to increase the risk of lung cancer in the Chinese Han population (Wang et al., 2015). Based on the above information, we predicted that PSMA4 may govern cell proliferation and apoptosis, and hence can promote the proliferation of lung cancer cells.

Additionally, HLA-DQB1, HLA-DRB9, and HLA-DQB2 are all homologs of HLA class II molecules, which are essential for immune response. HLA plays a key role in the interaction between tumor cells and the human immune system. HLA II molecules are involved in anti-tumor immunity and exogenous antigen presentation by CD4^+^ T cells. There is evidence that HLA II on tumor cells affects tumor migration and invasion, cancer progression, immune response and prognosis of a variety of malignancies *in vitro* and *in vivo*. For example, some studies have shown that tumor HLA-DQB1 level is associated with the recurrence free survival (RFS) of early Lung Adenocarcinoma, and the mechanism may be related to anti-tumor immunity (Zhang et al., 2019). Currently, there is no definitive study on the relationship between HLA-DRB9, HLA-DQB2 and lung cancer, and further verification will be needed.

CLPTM1L is a mitochondrial protein and is thought to be involved in lung cancer. Some studies have found that the expression of CLPTM1L is significantly increased in lung cancer tissues compared with normal tissues by immunohistochemistry, especially in lung adenocarcinoma (Ni et al., 2012). Also, the researchers found that increased copy number in the CLPTM1L region is the most common genetic event in the early stages of NSCLC (James et al., 2012). Some GWAS data illustrate that the region of CLPTM1L gene is associated with lung cancer. Therefore, the gene expression of CLPTM1L is closely related to lung tumor susceptibility.

Moreover, according to the GWAS results, the SNPs of RAD52 gene were associated with increased risk of lung cancer, particularly the development of lung squamous cell carcinoma (Shi et al., 2012). As the development of lung squamous cell carcinoma is closely associated with smoking, DNA repair is increased in the lung tissue of smokers (Lieberman et al., 2017). RAD52 encoded gene product can bind to the single stranded DNA terminal and can be involved in homologous recombination and DNA repair in mammals. To sum up, the relationship between RAD52 gene expression and the occurrence of lung cancer may be due to its DNA repair function in lung tissue.

The iron-binding protein encoded by IREB2 is involved in maintaining iron homeostasis in human cells. IREB2 is located in the susceptibility locus of lung cancer. A previous study showed that iron levels in the lungs increase with age, with higher concentrations in the lungs of smokers. Therefore, abnormal IREB2 expression or function may lead to iron metabolism disorder. Iron load leads to cell proliferation in cancer cell lines, and cancer cell lines have a poor ability to regulate IREB2 expression (Khiroya et al., 2017). In addition, GWAS results illustrate that IREB2 has been shown to be associated COPD and the expression of its protein product IRP2 is altered in lung cancer patients carrying lung cancer (Fehringer et al., 2012). The role of IRP2 is to control iron levels in cells by regulating the input, output and storage of various iron proteins. Therefore, the misexpression of IREB2 gene may alter intracellular iron levels and hence lead to the occurrence of malignant tumors. If IRP2 gene can be targeted therapeutically, it may provide a new approach for lung cancer treatment.

In summary, our study integrated genomic and transcriptome information and found seven genes associated with lung cancer by the SMR method, revealing possible regulatory mechanisms that alter the expression of these genes and further pathogenicity. This provides a research direction for understanding the precise treatment and targeted drug design of lung cancer. Although the SMR method can avoid the interference of confounding effect and reverse causal association in the study, and can be not limited by the sample size, thus producing more reliable results, this method can only preliminarily judge that the gene expression may affect the development of lung cancer, and cannot show how gene expression regulates the onset and progression of this disease. It takes experiments and other analytical methods to confirm a causal link between genes and disease. In the following study, we will continue to use SMR analysis method to integrate GWAS and other types of QTL data to mine more genes affecting lung cancer.

## Data Availability

The original contributions presented in the study are included in the article/Supplementary Material, further inquiries can be directed to the corresponding author.
